# A risk factor analysis of healthcare-associated fungal infections in an intensive care unit: a retrospective cohort study

**DOI:** 10.1186/1471-2334-13-10

**Published:** 2013-01-09

**Authors:** Su-Pen Yang, Yin-Yin Chen, Han-Shui Hsu, Fu-Der Wang, Liang-yu Chen, Chang-Phone Fung

**Affiliations:** 1 Division of Infectious Diseases, Department of Medicine, Taipei Veterans General Hospital, Taipei, Taiwan, No. 201, Sec. 2, Shih-Pai Road, Taipei, 112, Taiwan; 2 Department of Infection control and Department of nursing, Taipei Veterans General Hospital, Taipei, Taiwan, No. 201, Sec. 2, Shih-Pai Road, Taipei, 112, Taiwan; 3 Division of Chest Surgery, Department of Surgery, Taipei Veterans General Hospital, Taipei, Taiwan, No. 201, Sec. 2, Shih-Pai Road, Taipei, 112, Taiwan; 4 Institute of Emergency and Critical Care Medicine, National Yang-Ming University, Taipei, Taiwan, No.155, Sec. 2, Linong Street, Taipei, 112, Taiwan; 5 College of Nursing, National Yang-Ming University, Taipei, Taiwan, No.155, Sec. 2, Linong Street, Taipei, 112, Taiwan; 6 Institute of Public Health, National Yang-Ming University, Taipei, Taiwan, No.155, Sec. 2, Linong Street, Taipei, 112, Taiwan; 7 Center for Geriatrics and Gerontology, Taipei Veterans General Hospital, Taipei, Taiwan, No. 201, Sec. 2, Shih-Pai Road, Taipei, 112, Taiwan

**Keywords:** Intensive care unit, Fungal infection, Outbreak surveillance, Candida, Total parenteral nutrition

## Abstract

**Background:**

The incidence of fungal healthcare-associated infection (HAI) has increased in a major teaching hospital in the northern part of Taiwan over the past decade, especially in the intensive care units (ICUs). The purpose of this study was to determine the factors that were responsible for the outbreak and trend in the ICU.

**Methods:**

Surveillance fungal cultures were obtained from “sterile” objects, antiseptic solutions, environment of infected patients and hands of medical personnel. Risk factors for comparison included age, gender, admission service, and total length of stay in the ICU, Acute Physiology and Chronic Health Evaluation (APACHE) II scores at admission to the ICU, main diagnosis on ICU admission, use of invasive devices, receipt of hemodialysis, total parenteral nutrition (TPN) use, history of antibiotic therapy before HAI or during ICU stay in no HAI group, and ICU discharge status (ie, dead or alive). Univariable analysis followed by multiple logistic regression analysis was performed to identify the independent risk factors for ICU fungal HAIs and ICU mortality.

**Results:**

There was a significant trend in ICU fungal HAIs from 1998 to 2009 (*P* < 0.001). A total of 516 episodes of ICU fungal HAIs were identified; the rates of various infections were urinary tract infection (UTI) (54.8%), blood stream infection (BSI) (30.6%), surgical site infection (SSI) (6.6%), pneumonia (4.5%), other sites (3.5%). The fungi identified were: yeasts (54.8%), *Candida albicans* (27.3%), *Candida tropicalis* (6.6%), *Candida glabrata* (6.6%), *Candida parapsilosis* (1.9%), *Candida* species (0.8%), and other fungi (1.9%). *Candida albicans* accounted for 63% of all *Candida* species. Yeasts were found in the environment of more heavily infected patients. The independent risk factors (*P* < 0.05) of developing ICU fungal HAIs from all sites were TPN use, sepsis, surgical patients, mechanical ventilation and an indwelling urinary catheter. The independent risk factors for ICU fungal UTI included TPN use, mechanical ventilation and an indwelling urinary catheter. The independent risk factors for ICU fungal BSI included TPN use, sepsis, and higher APACHE II score. The independent risk factors for ICU fungal pneumonia included TPN use, surgical patients. The independent risk factors for ICU fungal SSI included surgical patients, and TPN use. The odds ratios of TPN use in various infection types ranged from 3.51 to 8.82. The risk of mortality in patients with ICU fungal HAIs was over 2 times that of patients without ICU HAIs in the multiple logistic regression analysis (*P* < 0.001).

**Conclusions:**

There was a secular trend of an increasing number of fungal HAIs in our ICU over the past decade. Patients with ICU fungal HAIs had a significantly higher mortality rate than did patients without ICU HAIs. Total parenteral nutrition was a significant risk factor for all types of ICU fungal HAIs, and its use should be monitored closely.

## Background

The incidence rate for invasive fungal infections has increased globally over the past 2 to 3 decades [1-3], especially in healthcare settings [4-6]. Along with this trend, these infections occurred more often in the non-neutropenic, critically ill patients [5,7-9] than in those patients who were neutropenic or had received organ transplantation in the past. *Candida* species comprised the majority of these pathogenic fungi [2,5,7]. In the United States (US), a national investigation of the epidemiology of sepsis from 1979 through 2000 reported that the rate of sepsis induced by fungi increased by 207 percent [1]. In the healthcare settings, *Candida* species were reported to be the seventh most common nosocomial pathogen in the National Nosocomial Infection Surveillance (NNIS) system conducted by Centers for Disease Control and Prevention (CDC) in the US during the 1980’s [10,11]. The incidence of nosocomial candidemia was also noted to increase substantially (219-487%) in both large and small hospitals at this time [12]. In a subsequent report using data from the NNIS system during the period of 1990 to 1992,*Candida* species were the sixth most common nosocomial pathogens from all sites and fourth most common pathogens in nosocomial blood stream infections (BSIs) [13]. When studies were limited to intensive care units (ICUs), *Candida* species were the fourth most common pathogens in ICU nosocomial infections (NIs) and BSIs in the 1980’s from the NNIS data [10,14]. Between 1992 and 1998, according to surveillance data collected from 205 combined medical-surgical ICUs in the US, *Candida* species and other fungi became the second most common pathogens in nosocomial urinary tract infections (UTIs) and BSIs [15].

In Taiwan, the burden of fungal healthcare-associated infection (HAI), formerly NI is also heavy, especially in the ICUs. A study from the Taiwan Nosocomial Infection Surveillance System (TNIS) reported *Candida* species to be the most common pathogen among the HAIs from 21 ICUs in 17 medical centers in 2009 [16]. According to this analysis, *Candida* species was the most common pathogen among the nosocomial UTIs, and the second most common pathogen among the nosocomial BSIs in these ICUs [16]. In one of these medical centers, *Candida* species and other yeasts had become the leading pathogens responsible for NIs hospital-wide since 1993, and *Candida* species were also the leading pathogens in nosocomial BSIs since with the highest rate in the medical ICU [5,17].

In our hospital, the overall incidence rate for fungal HAIs remained stable before 2006, although the incidence of candidal BSIs did increase in 2004, [18] however, the overall incidence of fungal HAIs rose abruptly in 2007, especially in the ICUs. The department of infection control initiated an outbreak investigation at the beginning of 2008. This included surveillance cultures and risk factor analysis for patients admitted to the ICU. Our aim was to determine if there was a common source, or if any specific factors were correlated with the trend of fungal HAIs. Although there have been reports of outbreak investigations or risk factor analyses for fungal HAIs, a complete study which includes risk factor analysis using a continuous prospective database along with an outbreak investigation is unique.

## Methods

### Hospital and setting

This study was conducted in a 42–bed adult medical–surgical ICU with more than 1500 admissions (age 18 years or older) per year located in a 2900–bed major teaching hospital in the northern part of Taiwan. Healthcare-associated infections, the onsets of which were more than 48 hours after admission to the ICU or within 48 hours after discharge or transfer to a ward, were considered to be acquired in the ICU [19,20]. The study was approved by the Institutional Review Board of Taipei VGH with a complete waiver of informed consent (2012-01-026BC).

### Nosocomial surveillance

A prospective hospital-wide surveillance for HAIs was established in 1982. A single infection-control professional surveyed the ICU during the entire period of this study [21,22]. All patients in the ICU were monitored for HAIs that affected particular body sites. The standard surveillance protocols and HAI site definitions, including BSI, UTI, surgical site infection (SSI), pneumonia and others sites, were those recommended by the CDC in the US [19,20,23]. Reports of HAI cases were also verified by an infectious disease specialist. Although the term “nosocomial” has been replaced by “healthcare-associated” infection since 2008, most of the case definitions remain unchanged.

### Definition of health-care associated infections

Nosocomial infections or HAIs are defined as a localized or systemic condition resulting from an adverse reaction to the presence of an infectious agent or its toxin. There must be no evidence that the infection was present or incubating at the time of admission to the acute care setting [19,23]. Thus, colonization should not be mistaken as NIs or HAIs by case definitions.

Symptomatic UTI was defined when a patient had one or more of the following signs or symptoms with no other recognized cause: fever (> 38°C), dysuria, urgency, frequency, or suprapubic tenderness and (1) the patient had a positive urine culture, that is, ≥ 10^5^ microorganisms per cc of urine with no more than two species of microorganisms, or (2) pyuria (urine specimen with ≥ 10 white blood cells/mm^3^) and a positive urine culture of ≥ 10^3^ and < 10^5^ CFU/ml with no more than 2 species of microorganisms. To detect UTI organisms, a urine sample was aseptically aspirated from the sampling port of a urinary catheter and cultured quantitatively [23]. Asymptomatic bacteriuria was removed from the case definitions of HAIs by CDC of the US in January 2009. Our department of infection control follows the modification of case definitions accordingly since January 2010.

Laboratory–confirmed BSI was defined when a patient had a recognized pathogen cultured from one or more blood cultures and the microorganism cultured from blood was not related to an infection at another site. Common skin contaminants (e.g., coagulase–negative staphylococcus [CoNS]) required culture from two or more blood cultures drawn on separate occasions or at least one blood culture for a patient with intravascular devices and microorganisms of the tip culture identical to those of the blood culture [23].

A SSI must meet the following criterion: Infection occurs within 30 days after the operative procedure, and the infection appears to be related to the operative procedure and involves soft tissues of the incision or infection involves any part of the body, and patient has at least 1 of the following: (1) purulent drainage from the deep incision or from the organ/space component of the surgical site (2) An incision spontaneously dehisces or is deliberately opened by a surgeon and is culture-positive or not cultured when the patient has at least 1 of the following signs or symptoms: fever (> 38°C), or localized pain or tenderness. (3) an abscess or other evidence of infection involving the incision is found on direct examination, during reoperation, or by histopathologic or radiologic examination. (4) organisms isolated from an aseptically obtained culture of fluid or tissue in the organ/space [23].

Pneumonia was defined when a patient had a new or progressive infiltrate, consolidation, cavitation, or pleural effusion on chest radiograph and had the following signs or symptoms: new onset of purulent sputum or change in character of sputum. To detect pneumonia microorganisms, tracheal aspirates obtained via endotracheal tube suction or tracheostomy tube suction were cultured [23].

### Data collection

In the combined ICU, data were collected prospectively for every patient who was admitted. The data collected for all patients included demographic characteristics (age, gender, admission service, and total length of stay in the ICU), Acute Physiology and Chronic Health Evaluation (APACHE) II scores at the time of admission to the ICU, main diagnosis on ICU admission, use of invasive devices (mechanical ventilator, central venous catheter (CVC), indwelling urinary catheter, arterial catheter, and wound drainage tube), receipt of hemodialysis, history of antibiotic therapy within 7 days before the HAI was identified or during the ICU stay in patients without HAI, and ICU discharge status (dead or alive). Information collected about each HAI included use of total parenteral nutrition (TPN) before HAI, the dates and sites of infection, and identified pathogens. Our microbiology laboratory identified fungi to the species level for most specimens except urine and sputum. For ordinary cultures of urine and sputum, fungal isolates were reported only as yeast or mold unless there was a special request. Identification of yeasts was performed by Vitek 2 YST card (bioMerieux Inc.).

The data about TPN use in ICU patients without HAI were provided by our TPN team from their prospective database. The TPN team is in charge of the consultation, preparation and quality control of TPN; and the members consist of physicians, dietitians, pharmacists and nurses. Prescription of antibiotics is monitored by the infectious disease specialists, and prophylactic antifungals are not recommended in the non-neutropenic, non-transplant ICU patients.

### Surveillance cultures

Surveillance cultures were performed from 16 March 2008 to 31 March 2008. Fungal cultures were obtained from the hands of all ICU healthcare workers including 10 physicians, 84 nurses, 2 administrative assistants, and 4 cleaning staff. Cultures were performed when personnel were off duty and after they had washed their hands. Cultures were also obtained from the environment of 11 patients with ICU fungal HAIs. These sites included tap water and drains, faucets, the surface of ventilators and monitors, patients’ drinking water, water used for sputum suctioning, bedside tables and bed rails. Fungal cultures were also obtained from “sterile” inanimate objects and disinfectants sampled from the ICU, respiratory care unit, neurological care unit, cardiovascular surgical unit, infectious disease ward and the supply center. These sterile inanimate objects included saline vials, sputum suction tubes, gauze, cotton swabs, urinary catheters, and urinary bag gel. All of these were sealed properly and never opened before cultures were obtained. Disinfectants included povidone iodine and antiseptic alcohol solution.

A sterile swab method and Sabaouraud dextrose agar were adopted for fungal cultures. The sterile swab was dampened with Sabaouraud dextrose broth when dry site cultures were performed such as on the surface of machines and hands of healthcare workers. All the specimens were incubated at 35°C for 14 days. The fungal isolates were then preserved in a −70°C refrigerator. Surveillance culture of TPN preparation is performed by the department of infection control as a routine at all times, including the whole study period.

### Statistical analysis

The *x*^2^ test was used to calculate a *P* value for the linear trend. To define an outbreak, the mean incidence rate of fungal HAIs from 1998 to 2006 in the ICU was used as the expected incidence before an abrupt increase was detected in 2007. An incidence exceeding the upper 99% confidence interval (CI) limit of the expected incidence was considered to be an outbreak.

By using the database described above, comparisons of all the collected variables were performed between patients with various types of ICU fungal HAIs and patients without ICU HAIs from January 2007 to April 2008. Descriptive analysis of all the collected variables was performed first. The *x*^2^ test was then used to evaluate the differences in categorical variables and independent t tests were used to evaluate the differences in numerical variables between the 2 groups. To identify independent risk factors that were associated with ICU fungal HAIs and ICU mortality, multiple logistic regression analyses were used to build up the models and adjust for confounders. All tests were 2-tailed, and *P* < 0.05 was considered to be statistically significant. All the analyses were performed using SPSS software, version 17.0 (SPSS).

## Results

**Figure 1:**
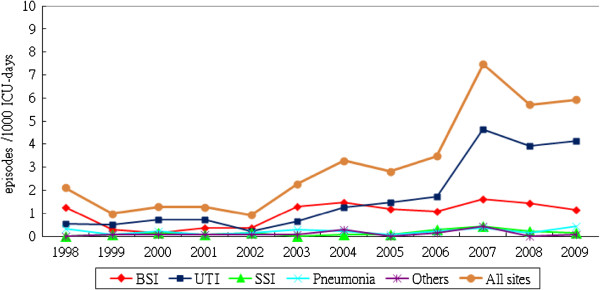
Trend for incidence of ICU fungal healthcare-associated infections from blood stream infection (BSI), urinary tract infection (UTI), surgical site infection (SSI), pneumonia, other sites and all sites.

The trend of fungal HAIs in the ICU from 1998 to 2009 is depicted in Figure 1. A total of 516 episodes of ICU fungal HAIs were identified; the rates of various infections were UTI (283, 54.8%), BSI (158, 30.6%), SSI (34, 6.6%), pneumonia (23, 4.5%), other sites (18, 3.5%). It appears that the rise in HAIs is parallel to the rise of UTIs, while BSIs increase to a much lesser extent. The *P* values for the linear trends of all sites, UTIs and BSIs were all < 0.001. The mean incidence of overall ICU fungal HAIs was 2.1 episodes per thousand person-days from 1998 to 2006, with an upper limit of 99% of CI 3.2. The incidence in 2007 was 7.5 episodes per thousand person-days, which was over 3 times the expected incidence. An outbreak of fungal HAIs in the ICU was suspected and an investigation was initiated. More strict infection control measures such as reinforcement of hand washing, auditing aseptic techniques and antibiotic prescription were adopted, and there seemed to be a tendency for decreased incidence after 2007, but the *P* value was not significant (*P* = 0.27).

### Outbreak surveillance

**Figure 2:**
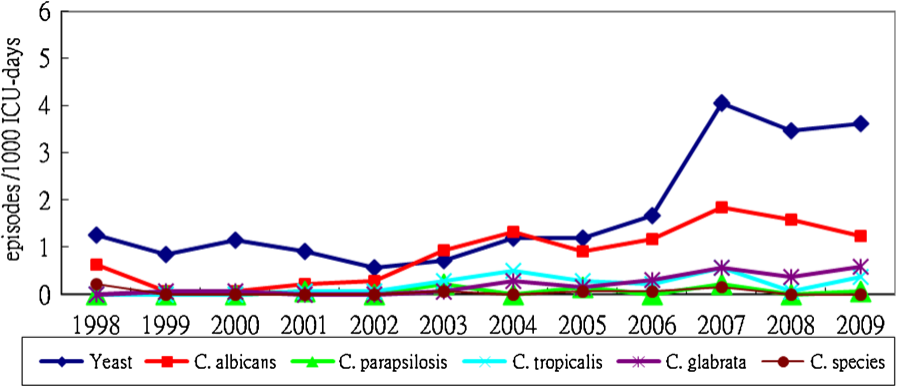
The species-specific distribution and trend for ICU fungal healthcare-associated infections from all sites.

The species-specific distribution and trend for all ICU fungal HAIs from 1998 to 2009 are depicted in Figure 2. A total of 516 fungal strains were isolated. The fungi identified were: yeasts (283, 54.8%), *Candida albicans* (141, 27.3%), *Candida tropicalis* (34, 6.6%), *Candida glabrata* (34, 6.6%), *Candida parapsilosis* (10, 1.9%), *Candida* species (4, 0.8%), and other fungi (10, 1.9%). *Candida albicans* accounted for 63% of all *Candida* species and it appears that that the rise in *Candida albicans* is parallel to the rise of BSIs in Figure 1. The rise in yeasts is parallel to the rise of UTIs in Figure 1. All of the various fungi showed a tendency for an increase of similar magnitude. No evidence of marked increase of a single species was observed.

Surveillance cultures of 39 samples from sterile objects and 47 samples from disinfectants were harvested. No fungal growth was detected in any of these specimens. Cultures yielded no fungal growth from the hands of any ICU healthcare worker either. During the whole study period, no TPN contamination event had been noted. Eleven patients were noted to have ICU fungal HAIs. Culture sites included urine, sputum, ascites, blood and pus. These patients were unrelated to one another in terms of unit location. Among them, 3 patients were noted to have 2 episodes of fungal HAI. In the first patient, the infection sites were UTI and CVC tip; yeasts were detected from the water used for sputum suctioning and the surface of the mechanical ventilator and the faucet. In the second patient with UTI and pneumonia infection sites, yeast was detected in the water used for sputum suctioning. In the third, 2 episodes of fungal UTI were noted and yeast was detected in his drinking water. In the remaining 8 patients with a single episode of fungal HAI, no fungus was detected in the environmental cultures.

### Patient demographic data

From January 2007 to April 2008, a total of 1674 patients admitted to the ICU had no ICU HAI; their mean age was 67.5 ± 18.0 years and 71.2% were male. The mean APACHE II score was 22.5 ± 7.6, and 305 patients died for a mortality rate of 18.2%. There were 186 patients with acquired ICU fungal HAIs; the rate of various infections were UTI (89, 47.8%), BSI (43, 23.1%), pneumonia (24, 12.9%), SSI (17, 9.1%), other sites (13, 7.0%). Their mean age was 71.6 ± 16.0 years and 70.4% were male. The mean APACHE II score was 24.7 ± 7.0, and 56 of these patients died for a mortality rate of 30.1%. The mortality rate was significantly higher in the fungal HAI group (*P* < 0.001). The proportion of TPN use was 2.6% in the non-HAI group and 19.4% in fungal HAI group; the average number of TPN utilization days before infection were 6.9 ± 6.7 vs 19.2 ± 1.7 days (*P <*0.001). The results of univariable analysis are shown in Table 1. Other variables with significant differences (*P* < 0.05) between the 2 groups included mean length of ICU stay, surgical service, diagnosis of digestive system disease, sepsis, prior antibiotic therapy, and use of invasive devices including a central line, mechanical ventilator, urinary catheter, wound drainage and hemodialysis.

**Table 1:** Demographic characteristics, univariable analysis between patients with ICU fungal HAIs and patients without ICU HAIs

**Variables**	**No HAI**	**Fungal HAI**	***p *value**
	**n = 1674(%)**	**n = 186(%)**	
Age, years mean ± SD,	67.5 ± 18.0	71.6 ± 16.0	0.001
APACHE II score, mean ± SD	22.5 ± 7.6	24.7 ± 7.0	<0.001
ICU stay before infection, mean ± SD	6.9 ± 5.7	9.8 ± 7.1	<0.001
Length of ICU stay, mean ± SD	6.9 ± 5.7	20.9 ± 14.7	<0.001
Total parenteral nutrition use before infection, days, mean ± SD	6.9 ± 6.7	19.2 ± 1.7	<0.001
Total parenteral nutrition use (yes)	44(2.6)	36(19.4)	<0.001
Gender (male)	1192(71.2)	131(70.4)	0.865
Service (surgical)	643(38.4)	105(56.5)	<0.001
Main diagnosis(yes)			
Neoplasms	356(21.3)	40(21.5)	0.939
Digestive system	334(20.0)	54(29.0)	0.003
Respiratory system	272(16.2)	21(11.3)	0.078
Genitourinary system	174(10.4)	14(7.5)	0.218
Sepsis	51(3.0)	13(69.9)	0.005
7days before fungal infection with antibiotic therapy	1042(62.2)	141(75.8)	<0.001
Mortality in ICU	305 (18.2)	56 (30.1)	<0.001
Invasive procedures (yes)			
Central venous catheter	1550(92.6)	178(95.7)	<0.001
Mechanical ventilator	1139(68.0)	148(79.6)	0.001
Urinary catheter	854(51.0)	123(66.1)	<0.001
Wound drainage	1550(92.6)	178(95.7)	<0.001
Hemodialysis	417(24.9)	65(34.9)	0.003

*SD*, Standard deviation; *APACHE*, Acute Physiology and Chronic Health Evaluation; *ICU*, intensive care unit; *HAI*, healthcare-associated infection.

### Risk factors

On multiple logistic regression analysis, significant risk factors for all sites and various types of ICU fungal HAIs after controlling for covariables are shown in Table 2. The independent risk factors (*P* < 0.05) of developing ICU fungal HAIs from all sites were TPN use (OR = 4.83, 95% CI, 2.80-8.34, *P* < .001), sepsis (OR = 2.71, 95% CI, 1.24-5.92, *P* = .012), mechanical ventilation (OR = 2.05, 95% CI, 1.40-2.99, *P* < .001), surgical patients (OR = 1.89, 95% CI, 1.27-2.80, *P* = .002) and an indwelling urinary catheter (OR = 1.52, 95% CI, 1.06-2.20, *P* = .025). The independent risk factors for ICU fungal UTIs were TPN use (OR = 3.51, 95% CI, 1.69-7.31, *P* = .001), mechanical ventilation (OR = 2.85, 95% CI, 1.73-4.69, *P* < .001), and an indwelling urinary catheter (OR = 1.64, 95% CI, 1.01-2.67, *P* = .045). The independent risk factors of developing ICU fungal BSIs were TPN use (OR = 8.47, 95% CI, 4.07-17.63, *P* < .001), sepsis (OR = 5.26, 95% CI, 1.70-16.27, *P* = .004) and higher APACHE II score (OR = 1.06, 95% CI, 1.02-1.11, *P* = .007). The independent risk factors for ICU fungal pneumonia were TPN use (OR = 8.07, 95% CI, 3.24-20.13, *P* < .001) and surgical patients (OR = 3.46, 95% CI, 1.44-8.28, *P* = .005). The independent risk factors for ICU fungal SSIs were surgical patients (OR = 11.79, 95% CI, 2.49-55.74, *P* = .002) and TPN use (OR = 8.82, 95% CI, 3.27-23.81, *P* < .001).

**Table 2:** Logistic regression analysis of risk factors for fungal infection

**Variables**	**Urinary tract infection**		**Blood stream infection**		**Pneumonia**		**Surgical site infection**		**All infection sites**	
	**OR**	**95% CI**	**OR**	**95% CI**	**OR**	**95% CI**	**OR**	**95% CI**	**OR**	**95% CI**
Age (>65 years)	0.90	0.54-1.50	0.70	0.30-1.34	1.45	0.58-3.62	0.58	0.23-1.48	0.94	0.64-1.40
Gender (male)	0.78	0.48-1.27	0.77	0.41-1.45	1.78	0.69-4.62	1.03	0.38-2.79	0.95	0.65-1.40
Service (surgical)	1.52	0.92-2.51	1.62	0.81-3.23	3.46^*^	1.44-8.28	11.79^*^	2.49-55.74	1.89^*^	1.27-2.80
APACHE II score	1.01	0.98-1.04	1.06^*^	1.02-1.11	1.03	0.97-1.09	0.99	0.93-1.06	1.01	0.99-1.04
ICU stay before infection(days)	0.98	0.95-1.01	1.00	0.97-1.03	1.01	0.99-1.03	0.99	0.95-1.04	1.00	0.98-1.02
antibiotic therapy	0.82	0.47-1.42	0.64	0.31-1.32	0.60	0.23-1.60	1.42	0.52-3.87	0.87	0.57-1.31
TPN	3.51^*^	1.69-7.31	8.47^*^	4.07-17.63	8.07^*^	3.24-20.13	8.82^*^	3.27-23.81	4.83^*^	2.80-8.34
Neoplasms	0.88	0.46-1.69	0.81	0.33-1.98	1.13	0.36-3.51	1.59	0.44-5.79	0.96	0.57-1.61
Respiratory system	0.90	0.43-1.88	0.60	0.18-2.03	1.06	0.26-4.33	—	—	0.79	0.42-1.47
Digestive system	1.03	0.53-2.01	1.21	0.51-2.88	1.84	0.60-5.60	2.61	0.78-8.72	1.32	0.80-2.19
Genitourinary system	0.76	0.32-1.81	1.00	0.32-3.11	1.20	0.30-4.79	—	—	0.72	0.36-1.44
Sepsis	1.60	0.56-4.57	5.26^*^	1.70-16.27	3.18	0.53-18.98	2.89	0.22-37.77	2.71^*^	1.24-5.92
Central line	2.63	0.61-11.24	0.90	0.20-3.99	0.51	0.11-2.46	6.80	—	1.21	0.50-2.92
Mechanical ventilator	2.85^*^	1.73-4.69	1.71	0.89-3.26	1.67	0.76-3.69	1.45	0.53-3.98	2.05^*^	1.40-2.99
Hemodialysis	0.70	0.36-1.37	0.72	0.29-1.80	1.67	0.62-4.47	3.08	0.87-10.81	1.21	0.74-1.98
Foley catheter	1.64^*^	1.01-2.67	1.02	0.56-1.88	2.00	0.92-4.35	0.92	0.37-2.33	1.52^*^	1.06-2.20
Wound drainage	0.76	0.29-2.02	1.52	0.56-4.12	0.15	0.02-1.43	1.01	0.29-3.55	0.96	0.49-1.88

*OR*, Odds Ratio; *CI*, Confidence interval; *APACHE*, Acute Physiology and Chronic Health Evaluation;

*TPN*, Total Parenteral Nutrition.

^*^: P value < 0.05; —: too few case numbers to calculate.

Predictors of ICU mortality were identified by multiple logistic regression analysis and are shown in Table 3. After adjustment for confounders, the risk of mortality in patients with ICU fungal HAIs was more than 2 times that of patients without ICU HAIs (OR = 2.16, 95% CI, 1.51-3.08, *P* < .001). Other predictors of ICU mortality included surgical patients (OR = 1.78, 95% CI 1.37-2.31, *P* < .001), a main diagnosis of digestive system disease (OR = 1.76, 95% CI 1.29-2.39, *P* < .001), sepsis (OR = 2.03, 95% CI 1.15-3.58, *P* = .014), neoplasm (OR = 1.83, 95% CI, 1.35-2.48, *P* < .001), and being on a mechanical ventilator (OR = 1.37, 95% CI, 1.05-1.81, *P* = .022).

**Table 3:** Predictors of ICU mortality

**Variables**	**Coefficients**	**SE**	**OR**	**(95% CI)**	***p *value**
Fungal HAI (yes/no)	0.77	0.18	2.16	(1.51-3.08)	<0.001
Service (surgical)	0.58	0.14	1.78	(1.37-2.31)	<0.001
Digestive system(yes/no)	0.56	0.16	1.76	(1.29-2.39)	<0.001
Sepsis(yes/no)	0.71	0.29	2.03	(1.15-3.58)	0.014
Neoplasms (yes/no)	0.60	0.15	1.83	(1.35-2.48)	<0.001
Mechanical ventilator (yes/no)	0.32	0.14	1.37	(1.05-1.81)	0.022

Results of multivariable analysis.

*ICU*, intensive care unit; *SE*, Standard error; *OR*, Odds ratio; *CI*, Confidence interval; *HAI*, healthcare-associated infection.

## Discussion

Our study began with an outbreak investigation of ICU fungal HAI. Nosocomial fungal outbreaks induced by a common exogenous source have been reported in the literature [11]. These have included contamination of milk bottles [24], parenteral nutrition [25], and IV medication [26]. Outbreaks induced by yeast carried on the hands of healthcare workers have also been reported [27-29]. There was no evidence of cross transmission or common source outbreak in our study. It was more likely a secular trend; however, yeasts were detected in the environment of more heavily infected patients. This finding still points out the importance of traditional infection control measures such as hand washing and environmental hygiene. Unfortunately, the fungal stocks of the environmental yeasts that were found failed to grow, so we cannot compare the species and genotypes of isolates yielded from the patients and their surroundings.

The second part of our study focused on an analysis of risk factors. Five independent risk factors were identified for all sites of ICU fungal HAI by the multivariable analysis, which included TPN use, sepsis, surgical patients, mechanical ventilation and an indwelling urinary catheter. These results are similar to those of previous studies, [30-34] though the case definitions of our study were different from others. Most of the previous studies defined cases by fungal growth from sterile or non-sterile body sites, or histology finding, or the EORTC/MSG criteria [34]. However, active surveillance on HAIs is a common practice in the hospitals worldwide, especially in the ICUs. The clinical implications from our study which defined cases with standardized HAI definitions can be closer to the daily practice in the ICUs.

Systemic review was performed by the Fungal Infection Risk Evaluation (FIRE) Study group to identify and summarize the important risk factors from published studies with multivariable analyses, risk prediction models and clinical decision rules for invasive fungal diseases (IFDs) in critically ill, adult patients. Meta-analysis was not performed due to the heterogeneity of these studies [34]. In this systemic review, the following risk factors were found in multiple studies to be significantly associated with IFD: surgery, TPN, fungal colonization, renal replacement therapy, infection/sepsis, mechanical ventilation, diabetes, APACHE II or III scores [34]. Five (surgery, TPN, mechanical ventilation, infection/sepsis, APACHE II score) of the 8 “important” risk factors reported by the FIRE Study group were also independent risk factors identified in various infection types of our study. The significant risk factors in our study were TPN use, mechanical ventilation and an indwelling urinary catheter in fungal UTI; TPN use, sepsis, and higher APACHE II score in fungal BSI; TPN use, surgical patients in fungal pneumonia; surgical patients and TPN use in fungal SSI. Total parenteral nutrition was a significant risk factor for all types of ICU fungal HAIs; the odds ratios were 3.51 in UTI, 8.47 in BSI, 8.07 in pneumonia, 8.82 in SSI, 4.83 in all sites infections.

The independent risk factor, patients with an indwelling urinary catheter, identified in UTI of our study had been reported in other studies; though they are not mentioned in the FIRE Study. A urinary catheter was included in Leon’s report from the EPCAN study group, but it was not a significant risk factor for proven fungal infection in comparison with the fungal colonization group [30]. They performed weekly surveillance cultures of urine, tracheal, and gastric samples, and compared risk factors between patients with unifocal or multifocal colonization and proven candidal infection. This may be explained by the fact that *Candida* species are frequently isolated from urine specimens from urinary catheters, whether symptomatic or not; especially when the patient is medicated with antibacterial agents [15]. The utilization rate of urinary catheters was high in both groups (colonized 98.5% vs. proven infection 95.9%) of Leon’s study, with broad spectrum antibiotics administered to 98% of the colonized vs. 100% of the proven infection group. In this case, a urinary catheter should be considered a common risk factor for both groups instead of an independent risk factor for proven fungal infection. In our study, the 2 groups for comparison were patients with ICU fungal HAI vs no HAI. Asymptomatic bacteriuria was still included as a specific infection type of healthcare associated UTIs during the study period according to the case definitions.

Diagnoses of sepsis, admission to the surgical ICU service and mechanical ventilation were significant risk factors for both ICU fungal HAIs and ICU mortality; these factors, especially sepsis and surgical patients, cannot be avoided in daily ICU practice. Early treatment had been documented to improve the outcome of candidemia, [35,36] although early diagnostic tools are still inadequate. Empirical or preemptive antifungal therapy has generally been accepted as a strategy under such circumstances; [37] therefore, when signs of ICU HAI appear; and more risk factors for fungal HAI exist, then stronger empirical antifungal therapy is indicated. Fungal ICU HAI was shown to be an independent predictor for ICU mortality in our study. Avoiding risk factors for ICU fungal HAIs, especially TPN use, may therefore improve ICU morbidity and mortality.

The mortality rate of patients with ICU fungal HAIs was noted to be over 2 times that of patients without ICU HAIs in our study. This was consistent with other reports in the literatures [30,38,39]. Candidemia has resulted in mortality rates as high as 50-70% [17,39-41]. Candiduria was documented to precede candidemia in the ICU, and itself had a mortality rate of 31.3% [40]. Fungal wound infections have been reported to be independently associated with mortality in burn patients [38]. These studies support our finding that patients with ICU fungal HAIs have a higher mortality rates than patients without HAIs regardless of the affected site. The rates and species distribution of fungal HAIs have differed geographically and institutionally, [9,42] and strains of *Candida* species resistant to antifungals have been increasing [43]. It is important for each healthcare unit to have its own epidemiologic knowledge about fungal HAIs in order to monitor the trend and formulate a strategy to control it.

Some limitations of our study should be noted. Firstly, our study was limited by the pre-existing database. Some factors listed in other studies such as corticosteroid use and the presence of chronic diseases were lacking. We did not specify chronic diseases among the variables because they had been included in the calculation of the APACHE II score. Corticosteroid use and chronic diseases as variables were not found to be significant risk factors in other studies [30,31]. Overall, most of the important risk factors were included in our study [34]. Secondly, the effort of environmental cultures from ICU patients was incomplete; because, surveillance cultures were not performed in patients with no HAI to see whether there were any differences. Finally, the information about the prescription of antibiotics was inadequate. The policy regarding the use of antibiotics may be correlated with fungal HAIs, and should be reviewed more comprehensively. However, no major new classes of antibiotics were introduced into our hospital during the study period. The prescription of antibiotics is complicated in the ICU; this could be the subject of a future study.

## Conclusions

There has been a secular trend of increasing fungal HAIs in our ICU over the past decade. Patients with ICU fungal HAIs had a significantly higher mortality rate than did patients without ICU HAIs. Total parenteral nutrition was a significant risk factor for all types of ICU fungal HAIs, and its use should be monitored closely.

## Abbreviations

US: United States; NNIS: National nosocomial infection surveillance; CDC: Centers for disease control and prevention; BSI: Blood stream infections; ICU: Intensive care unit; NI: Nosocomial infection; HAI: Healthcare-associated infection; UTI: Urinary tract infection; SSI: Surgical site infection; APACHE: Acute physiology and chronic health evaluation; CVC: Central venous catheter; TPN: Total parenteral nutrition; CI: Confidence interval; OR: Odds ratio; EORTC/MSG: European organization for research and treatment of cancer/invasive fungal infections cooperative group and the national institute of allergy and infectious diseases mycoses study group; FIRE: Fungal infection risk evaluation; IFD: Invasive fungal disease; EPCAN: Estudio de prevalencia de CANdidiasis Candidiasis Prevalence Study.

## Competing interests

The authors declare that they have no competing interests.

## Authors’ contributions

SPY contributed to the study concept, the execution of the study and manuscript preparation. CPF contributed to the study concept and design, the revision of the manuscript. YYC contributed to the study design, data acquisition and statistical analysis. HSH contributed to the study design and data acquisition. FDW contributed to data acquisition and manuscript preparation. LYC contributed to data analysis and tabulation. All authors read and approved the final manuscript.

## Authors’ information

SPY, Attending physician in Division of Infectious Diseases, Department of Medicine, Taipei Veterans General Hospital, Taipei, Taiwan; studying for master degree in Institute of Emergency and Critical Care Medicine, National Yang-Ming University, Taipei, Taiwan. CPF, Head of Division of Infectious Diseases, Department of Medicine, Taipei Veterans General Hospital, Taipei, Taiwan; Associate professor of Institute of Emergency and Critical Care Medicine, National Yang-Ming University, Taipei, Taiwan. YYC, Head nurse of Department of Infection control, Department of nursing, Taipei Veterans General Hospital, Taipei, Taiwan; Adjunct assistant professor of College of Nursing, National Yang-Ming University, Taipei, Taiwan. HSH, Head of Division of Total Parenteral Nutrition, Attending physician of Division of Chest Surgery, Department of Surgery, Taipei Veterans General Hospital, Taipei, Taiwan; Associate professor of Institute of Emergency and Critical Care Medicine, National Yang-Ming University, Taipei, Taiwan. FDW, Head of Department of Infection control, Attending physician in Division of Infectious Diseases, Department of Medicine, Taipei Veterans General Hospital, Taipei, Taiwan; Adjunct assistant professor of Institute of Public Health, National Yang-Ming University, Taipei, Taiwan. LYC, Attending physician in Center for Geriatrics and Gerontology, Taipei Veterans General Hospital; studying for master degree in Institute of Public Health, National Yang-Ming University, Taipei, Taiwan.

## Pre-publication history

The pre-publication history for this paper can be accessed here:

http://www.biomedcentral.com/1471-2334/13/10/prepub
